# Evaluation of Physical Fitness, Body Composition, and Adherence to Mediterranean Diet in Adolescents from Estonia: The AdolesHealth Study

**DOI:** 10.3390/ijerph16224479

**Published:** 2019-11-14

**Authors:** Pablo Galan-Lopez, Raúl Domínguez, Maret Pihu, Thordis Gísladóttir, Antonio J. Sánchez-Oliver, Francis Ries

**Affiliations:** 1 Physical Education and Sports, Faculty of Educational Sciences, University of Seville, 41013 Sevilla, Spain; pgalan1@us.es (P.G.-L.); fries@us.es (F.R.); 2 Faculty of Health Sciences of Universidad Isabel I, Universidad Isabel I, 09003 Burgos, Spain; raul_dominguez_herrera@hotmail.com; 3 Institute of Sport Sciences and Physiotherapy, Faculty of Medicine, University of Tartu, 51005 Tartu, Estonia; maret.pihu@ut.ee; 4 Research Center for Sport and Health Sciences, School of Education, University of Iceland, 105 Reykjavík, Iceland; thg@hi.is; 5 Human Motricity and Sports Performance, University of Seville, 41013 Seville, Spain

**Keywords:** adolescents, obesity, nutrition, sedentary, physical inactivity, lifestyle, cardiovascular risk factors, body composition

## Abstract

Unhealthy lifestyles, low levels of physical fitness, and adherence to the Mediterranean diet (MD) are associated with bad quality of life and the development of a wide range of non-communicable diseases (NCDs). The current study aimed to evaluate the level of adherence to the MD in physical fitness performance and body composition parameters in children and adolescents of Estonia. Therefore, 413 adolescents (56% boys) from the city of Tartu completed the Mediterranean Diet Questionnaire (KIDMED) for analyzing the adherence to MD and performed the Alpha Fitness Test for measuring physical fitness and body composition. A 41.67% of low, 44.05% of average, and 14.28% of high adherence to MD was detected, without difference between genders (*p* = 0.747). In the Alpha Fitness battery, a higher performance was observed in all tests for boys vs. girls (*p* < 0.05). In relation to body composition, higher height, weight, and waist values were observed in boys (*p* < 0.05) and a lower body fat percentage (*p* < 0.01) without differences in body mass index (BMI; *p* = 0.906). The adherence to the MD is classified as average/low. Gender significantly influences all variables of the Alpha Fitness battery and anthropometrics measures excepting BMI. According the levels of adherence to the MD, no statistically different prevalence was observed for Non-Overweight (N-O_weight_), Non-Overfat (N-O_fat_), or Non-Overwaist (N-O_waist_). Still, a risk factor for Overweight (O_weight_) in boys with low adherence was observed in comparison to those with a mid-level of adherence to the MD.

## 1. Introduction

The clinical manifestations of non-communicable diseases (NCDs) usually appear during adulthood. However, the development of the asymptomatic phase can start at an early age [1]. NCDs, also known as chronic diseases, tend to be long-lasting and are the result of a combination of genetic, physiological, environmental, and behavioral factors [2]. An unhealthy lifestyle can contribute to the development of cardiovascular diseases, the main NCDs in terms of premature deaths [3].

Obesity, a chronic disease of multifactorial origin, is one of the most serious and prevalent NCDs today. Its high prevalence worldwide (5.6% in girls and 7.8% in boys) is associated with potentially severe complications and requires a multidisciplinary approach due to its high clinical impact and healthcare costs [4]. Despite decades of struggle against this epidemic, which represents an important threat to public healthcare, the huge increase of obesity in childhood and adolescence is one of the greatest public health challenges of the 21st century [5], with the added problem of an unpromising future due to trends of growing rates [6,7].

The assessment and monitoring of health risks at these age stages are essential for the provision of resources and the planning of health services and preventive interventions [8], allowing the identification of the population groups and regions where the need may be greater [5]. Furthermore, these periods of life are very important due to the establishment of life habits and the large number of psychological and physiological changes that take place [9]. In both childhood and adolescence, health status is influenced by several factors, including dietary patterns and physical activity, which are fundamental elements of lifestyle in terms of primary prevention and therapeutic education [10]. Therefore, the habits adopted at these ages may represent an advantage or a risk for preventing NCDs, and they establish a good predictor of health and subsequent morbidity and mortality [10,11,12].

Malnutrition increases the risk of NCDs in childhood and adolescence [13], whereas a healthy dietary pattern such as the Mediterranean diet (MD) [14] is shown as a decisive factor in the protection against diseases and premature deaths [15,16], and has benefits for the treatment and the improvement of the most prevalent NCDs nowadays [2,14,16,17]. The MD is based on a high intake of fruits, vegetables, legumes, and unrefined cereals, a moderate to high intake of olive oil and fish (depending on the proximity to the sea), a moderate intake of milk, yogurt, and cheese, and a low consumption of meat or meat products [18]. The research on the adherence to the MD in children and adolescents from Mediterranean and non-Mediterranean countries and their influence on health has increased in recent years. This is due to its protective value, and as a treatment for NCDs such as cardiovascular diseases, diabetes mellitus type 2, or obesity, among others [19,20,21,22,23,24]. In addition, the food intake during adolescence, the fundamental age in the establishment of dietary patterns, is a significant predictor of food intake in adulthood [25].

Sedentarism and physical inactivity are two of the most determining factors of lifestyle and health in children and adolescents [26,27,28,29]. The WHO reports that more than 80% of adolescents do not meet the recommended levels of physical activity [30], whose benefits are widely documented [31,32], having a direct relationship with the prevention and treatment of NCDs at these ages [33,34,35,36]. The levels of physical fitness in adolescents are an important indicator of their lifestyle and seem to have positive consequences on health-related quality of life (HRQoL) [37]. A low level of physical fitness is associated with NCD risk factors that can persist into adulthood [38,39]. On the contrary, a high level of physical fitness in childhood and adolescence is considered essential for the maintenance of health, well-being, and a central defense against NCDs [40]. However, existing data indicate significantly low levels of physical fitness in children and adolescents [41,42]. Therefore, the control and the assessment of physical fitness is crucial for identifying and intervening in its modifiable factors, and thus setting up the appropriate public health strategies for these ages [43].

The current bibliography includes inverse correlations between NCDs, levels of physical fitness, normal range for body composition parameters values, and adherence to the MD in children and adolescents [15,16,44,45]. Furthermore, in turn, existing data relate an adequate body composition with good physical fitness and/or a high rate of adherence to the MD [14,16,17,46,47,48]. Considering the importance of the abovementioned factors, the purpose of this study was to evaluate the level of adherence to the MD in physical fitness performance and body composition parameters (body mass index (BMI), % of body fat and waist circumference) in 413 adolescents (boys and girls) from the city of Tartu (Estonia).

## 2. Materials and Methods

### 2.1. Study Design

The present study is a cross-sectional, descriptive, and quantitative research that has analyzed the adherence to the MD diet in body composition and physical fitness in Estonian adolescents. Deontological standards recognized by the Declaration of Helsinki (revision of Hong Kong in September 1989 and Edinburgh in 2000) were followed and the research was carried out in accordance to the recommendations of Good Clinical Practice of the EEC (document 111/3976/88 of July 1990). In addition, the Ethics Committee of the University of Tartu approved the present study (Ref.: 281/T-10).

### 2.2. Study Sample

The current research included 13 to 16-year-old school students from the city of Tartu (Estonia). To start, 588 participants (326 boys and 265 girls) were selected and, finally, 413 adolescents, 233 boys (56%, M_age_ = 15.05 ± 1.14 years) and 180 girls (44%, M_age_ = 15.17 ± 1.08 years) took part in it. The students were recruited from three randomly chosen schools in Tartu: Kivilinna Kool (43.8%), Forseliuse Kool (29.1%), and Tamme Kool (27.1%). The inclusion criteria for the current study were: Female and male subjects aged 13 to 16-years-old having submitted the signed informed consent by their parents or tutors. In relation to their health status, the participants were those students who attended Physical Education classes regularly. The participants had no cognitive, physical, or motor limitations. Adolescents were asked for verbal consent, being informed that their participation was voluntary and that they could withdraw from the research at any time.

### 2.3. Instruments

#### 2.3.1. Adherence to the MD (KIDMED)

Adherence to the Mediterranean Diet Questionnaire (KIDMED) was used to assess the adherence in adolescents (http://www.aulamedica.es/nh/pdf/9828.pdf) [49]. The questionnaire, previously validated, consists of 16 items, 12 of them suppose a positive score in relation to the adherence to the MD, and the other 4 suppose a negative score. Affirmative answers to the questions that involve greater adherence to the diet are worth +1 point. Affirmative answers to the questions that suppose fewer adherences to the diet are worth −1 point. KIDMED index is the result of the sum of the scores and ranges from 0 to 12 (minimum to maximum adherence). Adherence to the MD can be classified into three categories: Low adherence: Very low-quality diet (0 to 3 points); average adherence: Improvement of the dietary pattern is needed (4 to 7 points); and high adherence: Optimal MD (8 to 12 points).

#### 2.3.2. Alpha Fitness Test Battery

The extended version of the Alpha Fitness test battery (Ref: 2006120) was employed for assessing physical fitness and body composition. This battery consists of four tests: Handgrip strength test, long jump test, speed-agility, and cardiorespiratory fitness test [50].

The handgrip strength test, using a hand dynamometer with an adjustable fit (TKK 5401 Grip D, Takey, Tokyo, Japan), evaluates the maximum strength of upper extremities. Before the beginning of the test, an assessor showed the correct procedure and how to adjust the dynamometer individually in accordance to the size of the hand of every participant [51]. During the test, the assessor encouraged the participants verbally to “grip as strong as possible” in order to maintain the maximal strength for two seconds. The average result of the two attempts for each hand was recorded.

A long jump test from a standing position was performed in order to analyze the explosive strength of lower extremities. The participants were placed behind the jump line with both feet together realizing an explosive jump covering the longest distance possible. The distance was measured from the take-off line point where the back of the heel nearest to the take-off line landed on the ground. The best out of two jumps was recorded.

In order to measure the speed-agility, a 4 × 10 m test was employed. Before the start of the test, an evaluator showed how to run as quickly as possible the 10 m distance between two lines four times. In total, the participants completed two series. The participants realized the test twice being recorded the best time.

A 20 m shuttle test was performed for evaluating cardiorespiratory fitness [52]. During the test, the participants had to cover a distance of 20 m and back, adjusting their running pace to the rhythm of an external acoustic signal (beep). From 8.5 km/h, the running speed increased 0.5 km/h each minute due to a shorter time lap between the beeps. The test was finished when the participants were not able to reach anymore the line for the second time at the same time as the beep, or when they had to stop due to fatigue.

#### 2.3.3. Body Composition

The height of the participants was measured using a portable height rod with an accuracy of 0.1 cm (Seca 213, Seca, Hamburg, Germany). The subjects wore light clothing for measuring body weight (with an accuracy of 0.10 kg) and body fat percentage by a bioelectric impedance (Tanita Inner Scan BF-689, Tanita, Tokyo, Japan). The body mass index (BMI) was calculated from the ratio between body weight (kg) and the height squared of the participants (m^2^). The waist perimeter (with an accuracy of 0.1 cm) was measured with a non-flexible anthropometric tape measure (Seca 201, Seca, Hamburg, Germany). Waist circumference was measured at the level of the narrowest point between the lowest rib and the iliac crest at the end of a normal exhalation. In order to classify the participants attending to their body composition parameters, they were classified as Non-Overweight (N-O_weight_) or Overweight (O_weight_) (regarding their BMI), Non-Overfat (N-O_fat_) or Overfat (O_fat_) (related to their fat%), and Non-Overwaist (N-O_waist_) or Overwaist (O_waist_) (attending to their waist circumference) based on the reference values provided by Moreno et al. [53,54].

### 2.4. Methodology

The Alpha Fitness test battery and the KIDMED questionnaire were completed by all the participants during their physical education classes. The test battery was organized as a circuit and the different tests were performed successively. The cardiorespiratory fitness test was completed by several students at the same time on a different class day. The preparation and the implementation of all the physical tests took 90 min for each group of students.

### 2.5. Data Analysis

The qualitative variables are expressed in frequencies and percentages (%), while the quantitative variables are presented as means (M) and standard deviations (SD). The Kolmogorov–Smirnoff test was performed in order to verify the normality of the variables and the homogeneity of variances by Levene’s test. In order to analyze the adherence to the MD (low, medium, or high), the gender (boys and girls) and the interaction between gender∙adherence to the MD, a multivariate analysis of variance (MANOVA) was performed, taking as dependent variables the body composition parameters, physical fitness results, and KIDMED and Alpha Fitness index; gender and adherence to the MD were considered fixed factors. In the case of statistically significant differences, a Bonferroni post-hoc test was performed. Additionally, Eta-squared ($\eta_{p}^{2}$) was calculated with <0.25, 0.26–0.63, and >0.63 considered small, medium, and large effect sizes, respectively [55]. For analyzing the adherence to the MD in the prevalence of obesity attending to three criteria (O_weight_, O_fat_, and O_waist_), a Chi-square test was performed. Subsequently, an odd-ratio (OR) was calculated for estimating the risk of overweight based on the adherence to the MD. The level of statistical significance was fixed as *p* < 0.05. Data analysis was carried out using the SPSS statistical package (version 25.0, SPSS Inc., Chicago, IL, USA).

## 3. Results

### 3.1. Adherence to the MD (KIDMED)

No differences in the KIDMED index were found when comparing boys and girls (*p* = 0.673). When stratifying the whole sample by the level of adherence to the MD, we found that 41.67% of the participants showed low adherence, 44.05% an average adherence level, and 14.28% a high level.

### 3.2. Alpha Fitness Test Battery

Table 1 shows the physical fitness results in relation to gender and level of adherence to the MD in boys and girls. Statistically significant differences for gender can be observed in all physical fitness variables (*p* < 0.05), and no statistically significant results for the adherence to the MD or for gender·adherence to the MD (*p* > 0.05) were found. Regarding Alpha Fitness Test, the boys obtained statistically higher values than girls in handgrip strength (32.99 ± 0.50 vs. 25.92 ± 0.66, *p* < 0.001; $\eta_{p}^{2}$ = 0.152), jump (192.93 ± 1.99 vs. 169.26 ± 2.65, *p* < 0.001; $\eta_{p}^{2}$ = 0.107), cardiorespiratory fitness (6.75 ± 0.16 vs. 5.60 ± 0.21, *p* < 0.001; $\eta_{p}^{2}$ = 0.047), and speed-agility test (11.66 ± 0.09 vs. 12.21 ± 0.12, *p* < 0.001; $\eta_{p}^{2}$ = 0.031).

When categorizing the average levels of the boys and the girls into the different levels of physical fitness established by Ortega et al. [52], the values of the boys are classified as low in handgrip, medium in 4 × 10 m and resistance, and high in jump test. According to this categorization, the girls show high average values in all the physical fitness variables, except for handgrip, where the average value is classified as medium.

### 3.3. Body Compositions

Table 2 shows the relation between the adherence to the MD and gender regarding body composition parameters. Similarly, as physical fitness variables, statistically significant differences were observed for some of the variables analyzed only for gender (*p* < 0.05), with the exception of BMI, and no statistically significant results for the adherence to the MD and gender·adherence to the MD in any of the variables analyzed (*p* > 0.05). Statistically significant differences were found for gender, with higher values in boys vs girls for height (1.74 ± 0.09 vs. 1.65 ± 0.07, *p* < 0.001; $\eta_{p}^{2}$ = 0.996) and weight (66.2 ± 15.29 vs. 59.5 ± 11.77, *p* < 0.001; $\eta_{p}^{2}$ = 0.042). However, no significant differences were observed for BMI (*p* = 0.906; $\eta_{p}^{2}$ < 0.001). Moreover, lower values were found in boys vs. girls in body fat percentage (14.68 ± 0.47 vs. 25.21 ± 0.62, *p* < 0.001; $\eta_{p}^{2}$ = 0.310) and higher in waist (78.10 ± 0.66 cm vs. 71.07 ± 2.41 cm, *p* < 0.001; $\eta_{p}^{2}$ = 0.084).

In the analysis based on BMI (N-O_weight_ and O_weight_) (Table 3), a trend to significance was observed only in boys (*p* = 0.053), since it is a risk factor to have a low-level vs. mid-level of adherence to the MD (OR = 1.69 [1.03–2.79]). As for waist measurements (N-O_waist_ and O_waist_), a trend toward significant differences in boys (*p* = 0.098) was also observed, although there was no risk factor for O_waist_ based on the level of adherence to the MD. No statistically significant differences were observed in relation to the fat % (N-O_fat_, and O_fat_) for the total sample, boys and girls (*p* > 0.05). Moreover, no statistically significant differences for the total sample and for the girls in N-O_weight_ vs. O_weight_ and N-O_waist_ vs. O_waist_ were found. 

## 4. Discussion

The current research is the first to analyze the levels of adherence to the MD in adolescent school students in Estonia, and its associations with physical fitness and body composition in a sample of male and female adolescents. The data from the KIDMED questionnaire (low 41.6%, average 44.05%, and high adherence to the MD, 14.28%) provide notably lower results than those of studies conducted in Mediterranean countries [56,57], but similar to those of studies carried out in non-Mediterranean countries [19]. In turn, the results obtained in the current study match those found by similar studies in southern European countries [20,58,59], with a comparable percentage in subjects with a medium adherence to the MD, but with higher results in those who presented a low adherence to the MD. However, the adherence is inferior in comparison to the results of a recent study by Galan-Lopez et al., conducted in Icelandic adolescents, and where 14.99% of low adherence was found, 60.72% of mild adherence, and 24.29% of high adherence [24]. In agreement with the results obtained by Ozen et al., which exhibited important differences in relation to a high and a low adherence in the population analyzed, with a clear trend to abandon the MD [60], the results found in the current research show a tendency to decrease the patterns related to the MD, since the average and low adhesion quantify more than 85% of the participating population. In addition, as in previous studies, there is no difference in adherence to the MD according to gender [20,24,61]. Moreover, similar to different studies, there is no difference between levels of adherence to the MD related to gender.

When analyzing the results of the different physical tests of the Alpha Fitness battery by gender, it can be confirmed that there are differences in all the Alpha Fitness variables, with higher scores for the boys. These results are in line with those found in several studies with a similar population, in which a better performance was observed in the tests of handgrip strength, long jump, speed-agility, and cardiorespiratory fitness in boys with respect to girls [62,63,64,65]. In addition, in the analysis of body fat, a higher percentage of body fat was observed in girls (25.01% vs. 14.68%, *p* < 0.001), so the higher levels of lean body mass in the boys explain a higher ability to attain higher levels of strength, speed, and cardiorespiratory fitness [66], although this could also be due to a higher level of physical inactivity or sedentarism in girls, a fundamental determinant of NCDs [29]. Similar to the research conducted by Galán-López et al., the current research presents a novel aspect [24], since it compares the results of the tests of health-related physical fitness and level of adherence to the MD, seeking possible associations. Significant differences were observed in the performances in the tests, but there were no significant differences when relating the same result with the level of adherence to the MD, coinciding with what was found by Galan-Lopez et al. [61].

Once the variables of the body composition of the participants were calculated, significant differences were observed between boys and girls in weight, height, % of body fat, and waist circumference. These results are similar to those obtained in several studies with adolescent populations, in which the girls showed higher levels of adiposity, whereas the boys presented higher results in weight, height, and waist circumference [24,67,68,69]. Furthermore, it is necessary to highlight that, although there are differences in the variables of weight, height, body fat percentage, and waist circumference, the values of these variables, in addition to those related to BMI, are classified as average values based on the levels established by Moreno et al. [53,54]. Contrary to what was found in the AVENA study [70], the participants of the current research do not suffer from obesity, since the values of BMI, waist circumference, and percentage of body fat are classified as mean values. Furthermore, the data concerning the weight of the population are similar to the reference values for European adolescents provided in the HELENA study [71].

In contrast to what is reflected in several recent studies, which have found a direct relationship between adherence to the MD, weight, and BMI of the subjects [21,72], as well as waist circumference [73], our study does not show any association between the level of adherence to the MD and the body composition parameters.

On the other hand, there is growing evidence that health behaviors are grouped. During adolescence, a healthy diet combined with regular physical activity increases the likelihood of a healthy pattern of constant physical maturation [18]. In addition, there are independent and combined associations between physical fitness, physical activity, body composition, and adherence to the MD with HRQoL in children, adolescents, and adults [15,19,20,21,22], and with significant improvements in joint interventions [23,24,25].

A correct evaluation and control of physical activity, body composition, and dietary habits that are more determinant for health in these stages, is fundamental, even more so when the process in these ages is considered reversible [74]. In addition, habits can both prevent or reduce the risk factors associated with NCDs by implementing a healthy lifestyle [75]. The current research highlights the importance of developing and maintaining healthy physical fitness habits, as well as improving and maintaining dietary patterns focused on the MD, since, together, both result in a better HRQoL at these ages.

The cross-sectional design of the current study has limitations, since the conclusions should not be attributed to plausible causes, but they can be used as valuable indications to be taken into account for future research. In addition, the data collected (KIDMED) were self-reported, which could lead to an error in the reports and recall due to the nature of the study.

## 5. Conclusions

As a novel aspect, this article is the first to analyze the adherence to the MD in relation to the variables of physical fitness and body composition in Estonian adolescents.

The adolescents who participated in this research show medium levels of physical fitness, with higher results for the boys. The participants’ adherence to the MD is classified as average/low. Gender significantly influences all variables of Alpha Fitness battery and anthropometrics measures, except BMI.

No statistically significant difference in prevalence was observed for N-O_weight_, N-O_fat_, or N-O_waist_ in relation to adherence to the MD. Nevertheless, a risk factor for O_weight_ in boys with low adherence was observed in comparison to those with a mid-level of adherence.

## Figures and Tables

**Table 1:** Physical fitness results attending to gender and level of adherence to the Mediterranean diet (MD) in both boys and girls.

Variable	Gender	Low	Mid	High	*p*-Value Gender	$\eta_{p}^{2}$ Gender	*p*-Value Adherence to MD	$\eta_{p}^{2}$ Adherence to MD	*p*-Value Gender· Adherence to MD	$\eta_{p}^{2}$ Gender· Adherence to MD
Age (years)	Boys	15.18 ± 1.14	14.99 ± 1.16	14.95 ± 1.14	0.437	0.001	0.700	0.002	0.473	0.004
	Girls	15.13 ± 1.14	15.24 ± 1.07	15.06 ± 0.96						
Handgrip (kg/m^2^)	Boys	33.15 ± 0.74	32.42 ± 0.69	33.41 ± 1.11	<0.001 *	0.152	0.648	0.002	0.317	0.006
	Girls	24.83 ± 0.77	26.34 ± 0.79	26.60 ± 1.65						
Jump (cm)	Boys	191.11 ± 2.97	193.57 ± 2.75	192.50 ± 4.43	<0.001 *	0.107	0.193	0.008	0.605	0.002
	Girls	164.43 ± 3.07	172.75 ± 3.15	170.61 ± 6.60						
Speed-agility (s)	Boys	11.72 ± 0.14	11.69 ± 0.13	11.56 ± 0.21	<0.001 *	0.031	0.733	0.002	0.656	0.002
	Girls	12.27 ± 0.14	12.07 ± 0.14	12.29 ± 0.31						
Cardiorespiratory Fitness. (stages)	Boys	6.43 ± 0.24	6.88 ± 0.21	6.93 ± 0.34	<0.001 *	0.047	0.344	0.005	0.462	0.004
	Girls	5.46 ± 0.24	5.37 ± 0.25	5.97 ± 0.51						

Data expressed mean ± standard deviation (SD). * Significant difference for a factor; statistical significance at *p* < 0.05.

**Table 2:** Body composition measures attending to gender and level of adherence to the MD in both boys and girls.

Variable	Gender	Low	Mid	High	*p*-Value Gender	$\eta_{p}^{2}$ Gender	*p*-Value Adherence to MD	$\eta_{p}^{2}$ Adherence to MD	*p*-Value Gender· Adherence to MD	$\eta_{p}^{2}$ Gender· Adherence to MD
Height (m)	Boys	1.75 ± 0.09	1.74 ± 0.10	1.73 ± 0.10	<0.001 *	0.996	0.156	0.009	0.268	0.006
	Girls	1.65 ± 0.07	1.67 ± 0.06	1.63 ± 0.09						
Weight (kg)	Boys	67.47 ± 14.82	64.55 ± 14.40	67.67 ± 18.31	<0.001 *	0.042	0.896	0.001	0.154	0.009
	Girls	58.15 ± 9.51	60.89 ± 13.15	59.82 ± 14.60						
BMI (m/kg^2^)	Boys	21.83 ± 3.90	21.21 ± 3.54	22.44 ± 5.13	0.906	<0.001	0.305	0.006	0.397	0.005
	Girls	21.31 ± 3.52	21.84 ± 4.16	22.51 ± 4.81						
Fat (%)	Boys	14.37 ± 5.94	14.16 ± 5.97	15.53 ± 8.23	<0.001 *	0.310	0.582	0.003	0.600	0.003
	Girls	24.53 ± 5.79	25.61 ± 7.36	25.49 ± 8.46						
Waist (cm)	Boys	77.94 ± 8.93	77.19 ± 9.01	79.18 ± 12.73	<0.001 *	0.084	0.944	0.001	0.519	0.003
	Girls	70.84 ± 7.87	72.04 ± 8.82	71.42 ± 10.78						

Data expressed mean ± SD * Significant difference for a factor; statistical significance at *p* < 0.05.

**Table 3:** Classification of overweight and non-overweight in boys and girls with different level of adherence to the MD attending to different criteria.

Level of Adherence to MD	Gender	Body Mass Index					% Body Fat					Waist				
		N-O_weight_ (*n* = 301)	O_weight_ (*n* = 49)	*p*-Value			N-O_fat_ (*n* = 346)	O_fat_ (*n* = 67)	*p*-Value			N-O_waist_ (*n* = 306)	O_waist_ (*n* = 107)	*p*-Value		
				Boys	Girls	Total			Boys	Girls	Total			Boys	Girls	Total
Low	Boys	67.4% (60)	32.6% (29)	0.053	0.436	0.123	87.6% (78)	12.4% (11)	0.637	0.322	0.559	68.5% (61)	31.5% (28)	0.098	0.642	0.494
	Girls	75.9% (63)	24.1% (20)				81.9% (68)	18.1% (15)				78.3% (65)	21.7% (21.8)			
	Total	71.5% (123)	28.5% (49)				84.9% (146)	15.1% (26)				73.3% (126)	26.7% (46)			
Mid	Boys	80.8% (84)	19.2% (20)				90.4% (94)	9.6% (10)				79.8% (83)	20.2% (21)			
	Girls	72.2% (57)	27.8% (22)				75.9% (60)	24.1% (19)				72.2% (57)	27.8% (22)			
	Total	77.0% (141)	23.0% (42)				84.2% (154)	15.9% (29)				76.5% (140)	23.5% (43)			
High	Boys	65.0% (26)	35.0% (14)				85.0% (34)	15.0% (6)				65.0% (26)	35.0% (14)			
	Girls	61.1% (11)	38.9% (7)				66.7% (12)	33.3% (6)				77.8% (14)	22.2% (4)			
	Total	72.9% (37)	27.1% (21)				79.3% (46)	20.7% (12)				69.0% (40)	31.0% (18)			

Data expressed as percentages (%) and frequencies.
